# Life in Modern Asylums.—II

**Published:** 1889-05-04

**Authors:** 


### Life in Modern Asylums.—II.

We left our patient at the point where his symptoms had become so severe as to render asylum treatment desirable or necessary. If a case is likely to go so far, the sooner this is recognised, and asylum treatment commences, the better. The great value of early treatment is proved by the statistics of recovery in all asylums. I do not consider that the statistics do prove this. It is probably true, however, independent of statistics. And 99 per cent. of asylum men regard the statistics as proving it.

If at all possible, no deception as to his destination should be practised on the patient in being conveyed to an asylum. In many cases the patient has a foreboding that such a step will be thought necessary, and the whole truth may be told him. Often, when he does not admit that his mind is disordered, he will acknowledge that he is not well, and will agree to go from home for the sake of his health. Frequently his questions require no direct reply, but, if pressed, no direct mis-statement should be made. Obviously, to find that he has been deceived, makes a patient angry or apprehensive, and acts unfavourably on his mental condition.

Our patient, now—let us hope with as little compulsion as possible—has arrived at the doors of the asylum, and been placed in a waiting-room. Here his treatment may be said immediately to commence. One or more attendants quietly take the place of his former inexperienced companions. Their quiet demeanour and firm, tactful manner reassure the timid and tend to restrain the unruly. The medical officer, who shortly appears to interview the newcomer and give orders for his admission, is also quiet and unflurried by a scene to which he is well accustomed. Questions are asked of the patient and those accompanying him in a systematic but kindly manner, which shows the patient he is an object of interest, while an apparent disregard of his erratic words and actions may for the first time make him feel doubtful of their propriety.

It is an almost universal practice to weigh patients on admission. This is generally done in the waiting-room, and is, when the reason is explained, often readily assented, or, at least, passively submitted to. On refusal, however, the excited or delusional patient will find himself quietly lifted on to the scale by two or more impassive attendants. This will be his first encounter with the physical force which must always exist in asylums, but which is carefully kept in the background. Few patients are so entirely devoid of reason as to be unable to draw the simple deduction that reasonable requests are going to be firmly enforced by his new neighbours. The use of physical force at the weighing process is therefore not unfrequently the last as well as the first occasion its employment is necessary. But there will have been no wrangling dispute, nor any exultation over their victory on the part of the asylum staff. If conscious of his surroundings, the new patient thus receives on the threshold of the asylum valuable moral treatment. He finds himself among people whose kindly words are backed by quiet firmness. No harm seems likely to be done him, but, on the other hand, he sees he will not necessarily get entirely his own way.

The papers of committal, etc., having been meantime examined and found correct, he will be passed into the wards. At this point he parts with his friends, and for the latter this is naturally a painful scene. For the patient himself, in the great majority of cases, this parting is no great trial. Frequently he is not fully conscious of its meaning, and in nearly all cases of mental disorder the affections are greatly blunted, in some cases positively perverted. This sad symptom may be better understood when one considers the fact that the rational emotions of family affection, etc., are among the highest functions of the brain, and thus among the first to be affected by any disorder of its working.

A warm bath is usually given to new patients. It is especially necessary in those of a class not generally very cleanly in person; and where a bath on admission is the rule, and the patient is not considered by the medical officer too weak to bear it, it is insisted upon. Here again, as at the weighing process, any unreasonable resistance offered is overcome by judicious firmness. The patient is examined while at the bath that any bruises or unusual external appearances may be at once detected, and he is thereafter placed in bed. The warm bath, besides its obvious use, is a valuable sedative, and its soothing effect may aid in bringing on natural sleep in an excited patient who has had no such sleep for days and nights previous to admission.

After being placed in a warm bed, a medical examination is usually made without delay, that any injuries or bodily diseases may be at once discovered. The patient here finds himself a person of importance, and sees that he is evidently going to be thoroughly looked after. At this point food will be offered, and even pressed upon him. Often it is thankfully accepted. The simple offer of a cup of tea to a frightened and exhausted female patient newly arrived, may make her a friend of the doctor for life.

The new comer is now fairly ensconced in bed, the initial formalities are over, and he finds himself left under the eye of an experienced attendant, but not otherwise interfered with. If violent, he may have to be quietly held in bed by one or more attendants, and some composing draught may be necessary to prevent his exhausting himself by his struggles. But in most cases, even if considerably excited beforehand, quieted by the moral treatment he has already received, by the warm bath and the food, and soothed by finding himself at rest in the bed after his journey, he is content to lie quiet and take what stock he can of his surroundings.

We have a great belief in the value of a day or two in bed for new patients. There, not only can they best be watched and medically examined, but the rest this treatment affords is almost always required. Moreover, from his bed, as a sort of coign of safe vantage, the timid new-comer can safely and quietly take note of his surroundings, and gain some acquaintance with the character of his neighbours before he has to rub shoulders with them in the day-rooms.

Our patient's bed will be placed in a room where he will pass the night with other patients and with experienced night attendants, who will watch and note his conduct for the information of the medical officers, and who will readily comply with his reasonable requests, and quietly but judiciously ignore any unreasonable acts or conversation. A certain well-judged amount of what may appear to a patient neglect is of much value in treating the very assertive or constantly complaining and unreasonably discontented. The room will be properly warmed and ventilated, and will not be left in darkness. If necessary, a composing draught will be given at bedtime; but most asylum physicians judge it best, as a rule, to give a chance to natural sleep, and take the opportunity of observing the tendencies of the patient for at least the first night. It must not be supposed that all excitement is to be quelled by narcotics. The peace thus purchased would in many cases be indeed a false peace, and as injurious to the individual patient as it is well known the abuse of narcotics is to any ordinary person. Some patients, it is true, would die of exhaustion if their violence was not restrained by sedatives, but in many large asylums many consecutive nights will pass in which not a single sedative draught is given to any patient. "Chemical restraint" is the name that has been given to the abuse of the sedative effect of narcotics in asylums. It is needless, we hope, to say that the interests of the individual patient, with, to a lesser extent, the comfort and safety of his fellow-patients, are the only indications for the use of drugs followed by a conscientious asylum physician.

Our patient's short experience of asylum life has already touched several important points of his new treatment, and we may leave him for the present with a fairly confident hope that, with or without a composing draught, he will pass a better night than for some time previous to admission.

